# A new and improved algorithm for the quantification of chromatin condensation from microscopic data shows decreased chromatin condensation in regenerating axolotl limb cells

**DOI:** 10.1371/journal.pone.0185292

**Published:** 2017-10-12

**Authors:** Julian Sosnik, Warren A. Vieira, Kaitlyn A. Webster, Kellee R. Siegfried, Catherine D. McCusker

**Affiliations:** 1 Department of Biology, University of Massachusetts Boston, Boston, Massachusetts, United States of America; 2 Department of Interdisciplinary Engineering, Wentworth Institute of Technology, Boston, Massachusetts, United States of America; Pennsylvania State Hershey College of Medicine, UNITED STATES

## Abstract

The nuclear landscape plays an important role in the regulation of tissue and positional specific genes in embryonic and developing cells. Changes in this landscape can be dynamic, and are associated with the differentiation of cells during embryogenesis, and the de-differentiation of cells during induced pluripotent stem cell (iPSC) formation and in many cancers. However, tools to quantitatively characterize these changes are limited, especially in the *in vivo* context, where numerous tissue types are present and cells are arranged in multiple layers. Previous tools have been optimized for the monolayer nature of cultured cells. Therefore, we present a new algorithm to quantify the condensation of chromatin in two *in vivo* systems. We first developed this algorithm to quantify changes in chromatin compaction and validated it in differentiating spermatids in zebrafish testes. Our algorithm successfully detected the typical increase in chromatin compaction as these cells differentiate. We then employed the algorithm to quantify the changes that occur in amphibian limb cells as they participate in a regenerative response. We observed that the chromatin in the limb cells de-compacts as they contribute to the regenerating organ. We present this new tool as an open sourced software that can be readily accessed and optimized to quantify chromatin compaction in complex multi-layered samples.

## Introduction

Changes to the nuclear landscape in cells are hallmark of many developmental processes occurring in embryos, adults, and diseases such as cancer. These changes, in part, are dependent on the spacing of the most basic component of chromatin known as the nucleosome. The nucleosome is comprised of a core of double stranded DNA wound around an octomeric histone complex, with each of the nucleosome core particles linked to the next by “linking DNA” [1,2]. Epigenetic modifications that occur to these nucleosomal particles controls their spacing, which regulates the density of the chromatin compaction, and the ability of transcriptional machinery to access the DNA (as reviewed by [3]).

Epigenetic modifications to the nucleosomes, in turn, can result in large-scale changes to the nuclear architecture (as reviewed by [3]). For example, increased abundances of tri-methyl marks on lysine residue number 27 of the nucleosomal core protein histone 3 (H3K27-me3) results in gene silencing and can lead to large-scale compaction of the DNA in these regions [4–6]. Examples of these large-scale changes occur frequently during cellular differentiation (as reviewed by [7], [8]), and have been largely characterized quantitatively in tissue culture cells [9]. There are a variety of different methods available to assess this molecular phenotype, including flow cytometry and DNase I hypersensitive site cleavage, however, these techniques are associated with a loss in spatial resolution during the analysis. Only a few techniques that quantitatively assess the changes in chromatin compaction *in situ* have been published. These methods utilize tissue culture cells and evaluate nuclear architecture quantitatively through changes in DNA density within the nucleus, assessed through spatial differences in local intensities of DNA specific dyes using fluorescence microscopy or fluorescence lifetime microscopy [10,11]. While the fluorescence lifetime microscopy technique developed by Spagnol and Dahl is compelling, it requires specialized equipment that is not readily accessible. On the other hand, the technique described by Irianto *et al*. uses standard fluorescent microscopy that is far more available. The general homogeneity of the cultured cells quantified, with roughly equivalent nuclear size and spacing, and the ease at which images can be obtained are strengths of this assay. However, methodological and computational issues are present in the technique proposed by Irianto *et al*. that makes it inapplicable in cases where the observations are made in complex multi-layered tissues composed of more than one cell-type. The focus of the current project was to establish a rigorous method to quantify the nuclear architecture *in situ*, which could be applied to different species and developmental processes.

We initially sought to quantify changes in nuclear architecture using a well-characterized system, where such alterations have qualitatively been observed before. Since large-scale chromatin condensation is known to occur during spermatogenesis, we decided to apply our method to this process in adult zebrafish testes.

In order to apply the method by Irianto *et al*., to complex tissues *in situ*, we modified the methodology for establishing thresholding levels. To assess chromatin compaction in cell culture, Irianto *et al*. established thresholding values for the images independently from each other, including and excluding data from the images in an individual fashion. While tissue culture cells would behave in a similar way upon treatment and staining, this is not the case of complex multi-layered tissue. Thus, we needed a method that would establish thresholding levels considering the whole dataset. To further improve the methodology, as to maximize the information within the data, we modified the algorithm to analyze 16-bit images. The published algorithm by Irianto *et al*. is optimized for 8 bit images yet most microscopes today utilize 12 or 16-bit imaging devices. The compression of 12 or 16-bit images to 8 bits implies loss of data. We wanted to develop an alternative that would permit loss-less (compression-less) image analysis that is both fast and reliable. Here we present an improved algorithm that performs chromatin condensation quantification from fluorescent microscopy data that applies thresholding values calculated for the entire dataset being analyzed, and calculates chromatin condensation indices in a rapid and robust fashion using uncompressed microscopy data as big as 2048 by 2048 pixels at 16-bits of depth.

Last, we applied our new algorithm to the nuclei in salamander cells that were undergoing the process of limb regeneration. These cells have been qualitatively recognized to undergo large-scale modifications to the nuclear architecture [12], but have not been evaluated through rigorous quantitative methods. We discovered that a quantifiable and significant de-condensation of the chromatin occurs in mature cells as they contribute to the regenerating salamander limb.

## Materials and methods

### Animal husbandry and surgeries

The Mexican axolotls (*Ambystoma mexicanum*) (RRID: AGSC_101J) used in this study were either spawned at the University of Massachusetts, Boston or obtained from the Ambystoma Genetic Stock Center, University of Kentucky. This study was carried out in accordance with the recommendations in the Guide for the Care and Use of Laboratory Animals of the National Institutes of Health. The experimental work was approved by the IACUC of the University of Massachusetts, Boston. For all surgeries, animals were anesthetized using a 0.1% solution of MS222 (Ethyl 3-aminobenzoate methanesulfonate salt, Sigma), pH 7.0. Regeneration was initiated by amputating the axolotl forelimb in the mid-humerus. Tissue samples were collected seven days post amputation, when the regenerating blastema had reached “early-blastema” stage. Zebrafish (*Danio rerio*) (RRID: ZIRC_ZL1) were maintained by standard conditions. Institutional IACUC approval was attained prior to carrying out all animal procedures. Zebrafish were sacrificed by exposure to 0.004% MS222 until opercle movement ceased; tissue was then harvested.

### Sample preparation

Torsos of adult male zebrafish were fixed in 4% paraformaldehyde (PFA) overnight, processed with methanol, and then decalcified in 0.5M EDTA pH 8 for 4 hours at room temperature prior to embedding in OCT for cryosectioning. Harvested axolotl limb tissues were fixed overnight at 4°C in 4% PFA, decalcified in 0.27M EDTA at room temperature for 2 days; and incubated in 30% sucrose overnight at 4°C; equilibrated and flash-frozen in OCT compound (Tissue-Tek). Sections were cut at 10 microns.

### Tissue staining

Axolotl and zebrafish tissue sections were incubated twice, at room temperate, with PBS for 5 minutes to remove the residual OCT compound, and then with 2.5μg/ml 4',6-diamidino-2-phenylindole dihydrochloride (DAPI, Sigma) for 15 minutes at room temperature in a dark chamber. Tissue sections were washed four times with 0.1% Tween in PBS and then mounted with Vectashield antifade mounting medium (Vector Laboratories).

### Microscopy

Fluorescent images were obtained using a 63x, 1.4NA Plan-APOCHROMAT oil immersion objective in a Zeiss Axio-observer Z1 inverted fluorescent microscope equipped with an Apotome 2 structured illumination optical sectioning device, and a Hamamatsu Orca 4 Flash LTE cooled monochrome camera. Z-stacks (with an optimal slicing distance of 0.24μm) were obtained of the mature dermal (at least 500μM away from amputation surface) and regenerating blastema tissue mesenchyme, and zebrafish testes.

### Data analysis

We develop a new algorithm and MATLAB (MathWorks, Natick, MA) code to perform quantitative chromatin condensation analysis of large datasets of 3-dimensional images in an automated and robust fashion (S1 Fig). The program (in different versions) and instructions on how to use it are feely available online (https://mccuskerlab.github.io/ChromCon/). The main principle underlying our analysis is that condensed chromatin delineates different nuclear domains [7]. These domains can be observed *in situ* via fluorescence microscopy by staining with DAPI (4’, 6’-diamidino-phenylindole)][13].

#### Chromatin condensation quantification

Our analysis algorithm (S1 Text), based on the one published by Irianto *et al*. [10] utilizes Sobel edge detection [14] to determine the areas of condensed chromatin within the nuclei of each cell. To perform consistent analysis on sets of 3-dimensional images without biasing such analysis by thresholding artifacts, our method first generates a unified dataset that consists of every non-zero pixel of the entire image set to be analyzed and establishes a thresholding level that will be later applied to all the images equally. This step allows us to perform quantitative analyses in all the z-planes of all the images to be analyzed individually while preserving the equivalency of levels that is required for comparative studies of data from the different conditions being studied.

Edge detection algorithms, like the Sobel detection we used, are susceptible to artifacts due to high frequency noise. To minimize this, we applied a Gaussian blur to the images being quantified [15] because it minimizes high frequency noise while preserving the edges unlike average filters. This allowed us to generate a robust algorithm that did not require pixel reduction or intensity redistributions, thus preserving the original data from the images. Large images without pixel reduction can result in increased running times for the analysis. Further optimizations on the algorithm, however, produced reliable results at faster rates than those from the existing algorithm (see results and discussion).

The nuclei were segmented and the remaining internal holes were filled, before extracting them to a black background. The nuclear area was calculated and the edges were detected and quantified in the thresholded images. The program then saved the data as comma separated values (csv) files that combined the data from the whole image stack in a single csv file saved to the same folder that the image originated from. These steps were then repeated for all the folders containing image stacks.

#### Pre-analysis image processing

To generalize our analysis tool and extend it to all platforms, independently of the format in which the images were originally saved or exported, we first developed a macro for ImageJ [16] that can crop images in 3 dimensions, in a lossless fashion, and separate z-stacks into single z-plane images in a unified folder for each color channel of the original image (S2 Text). The folders containing the nuclear stain channel can then be made into sub-folders of a general dataset folder that will be the input for our MATLAB analysis (S2 Fig). The macro also creates a single cropped stack for each color that can be used for additional analysis with other tools or algorithms. This macro and instructions for its use are distributed freely online (https://mccuskerlab.github.io/ChromCon/). This macro was utilized to segment in average 22 nuclei per sample that were then subject of quantification using the MATLAB analysis tool.

## Results and discussion

### Methodological improvements and algorithm efficiency

As mentioned above, computational and methodological issues hindered the utilization of the algorithm developed by Irianto *et al*. to quantify chromatin condensation *in situ*. The first problem that needed to be solved was the independent thresholding of the data. To prevent biasing in our analysis and retain comparable datasets, our algorithm takes into consideration the entirety of the dataset to be analyzed and performs only a single thresholding calculation for all the data collectively. This assures that the threshold levels are applied evenly throughout the entire sample set rather than individually thresholding each image. This generates analysis data that can be accurately compared within datasets.

**Fig 1:**
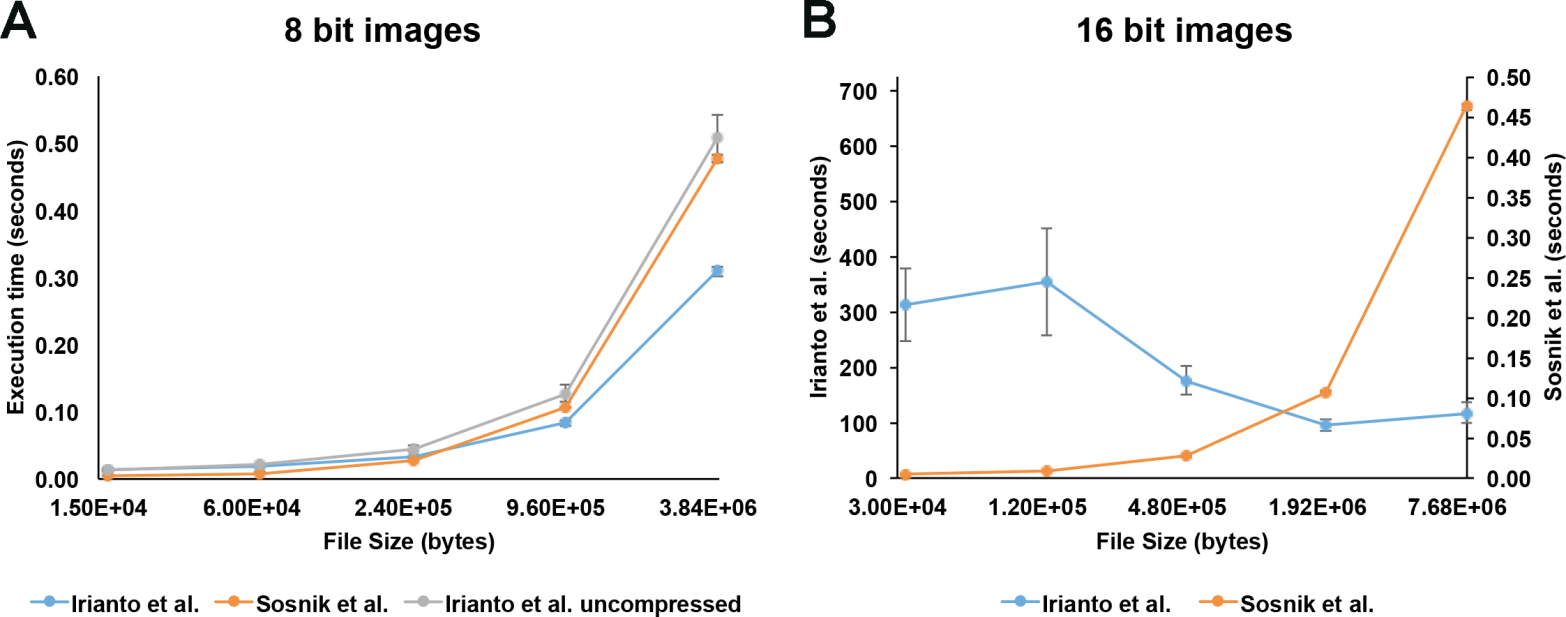
Improved computational efficiency of new chromatin quantification algorithm. Plots represent the amount of time (in seconds) it takes for the new algorithm (Sosnik *et al*.) and the previously published algorithm (Irianto *et al*.) to process different amounts of data. (A) Images ranging from 2048 by 2048 pixels to 128 by 128 pixels in 8 bits of data depth were analyzed with both algorithms. The Irianto *et al*. was tested using the reported algorithm (Irianto *et al*.) and a modified algorithm in which we eliminated the pixel reduction (compression) (Irianto *et al*. uncompressed). n = 15 images per size. Bars represent standard error. Analysis of variance followed by Tukey’s multiple comparison tests revealed significant to very significant differences (p < 0.05–0.01) between the Sosnik *et al*. algorithm and the Irianto *et al*. algorithm for small images (128–512). The comparison of the Sosnik *et al*. algorithm and the Irianto *et al*. uncompressed algorithm for larger images was not significantly different. (B) Images ranging from 2048 by 2048 pixels to 128 by 128 pixels in 16 bits of data depth were analyzed with both algorithms. n = 15 images per size. Bars represent standard error. Student t test for each image size revealed very to extremely significant (p = 0.0028 - <0.0001) differences between the Sosnik’s and Irianto’s algorithms.

Additionally, the methodology implemented to automatically calculate threshold levels in the Irianto *et al*. algorithm is recursive; meaning that it attempts to generate a threshold level and if it cannot, it applies a filter to the image and tries again. This step is repeated over and over until it either generates a threshold value or fails to establish one after 10,000 iterations. Recursive approaches like this one are time consuming and much less efficient than other alternatives that make a singular calculation. Our new algorithm utilizes Otsu’s method for non-parametric thresholding of gray levels [17]. This non-recursive method is capable of establishing maximal separation of gray levels through discriminant analysis of the image’s gray level histogram. Because of MATLAB’s robust implementation of Otsu’s method, our algorithm achieves highly discriminant levels from 16-bit images in a fraction of the time that the recursive method utilizes. In order to test this, we timed the execution times of both algorithms with images or various sizes (2k^2^ to 128^2^ pixels) at two bit depths (8 and 16 bits) (Fig 1 and Table 1). While the two methodologies seem to be comparable in execution time at 8 bits (with Irianto’s method appearing to run up to 50% faster with 2K^2^ images), it is important to consider that the published algorithm bins the image, effectively analyzing an image that is 4 times smaller than what was imputed. If we eliminate this compression and compare the execution times of images of equal size (Fig 1A, Irianto *et al*. uncompressed), our new algorithm is between 6 and 61% faster (Fig 1 and Table 1). Additionally, comparison of the two algorithms ability to analyze 16-bit images reveals that our new algorithm executes between 255 and 62500 times faster than the previous method (Fig 1 and Table 1).

**Table 1:** Efficiency and robustness of the different algorithms.

		Irianto *et al*.		Sosnik *et al*.		Irianto *et al*. uncompressed	
	File size (bytes)	Exec. Time(s)	Robustness(%)	Exec. Time(s)	Robustness(%)	Exec. Time(s)	Robustness(%)
**8 bit**	1.64E+04	0.0139	73.33%	0.0047	100.00%	0.0122	73.33%
	6.55E+04	0.0193	86.67%	0.0090	100.00%	0.0219	86.67%
	2.62E+05	0.0320	66.67%	0.0260	100.00%	0.0450	66.67%
	1.05E+06	0.0832	40.00%	0.1063	100.00%	0.1269	40.00%
	4.19E+06	0.3096	53.33%	0.4768	100.00%	0.5071	53.33%
**16 bit**	3.28E+04	312.6079	80.00%	0.0050	100.00%	309.0418	80.00%
	1.31E+05	353.9429	86.67%	0.0092	100.00%	349.5005	86.67%
	5.24E+05	175.6697	53.33%	0.0268	100.00%	172.8219	53.33%
	2.10E+06	95.6023	20.00%	0.1068	100.00%	94.3313	20.00%
	8.39E+06	117.9900	0.00%	0.4624	100.00%	116.0167	0.00%

Another observation that arose from our comparison analysis was the high level of failure that occurred when using Irianto *et al*.’s algorithm, in particular with images of large size. As published, the Irianto *et al*. algorithm has a limit in the recursive thresholding method that causes it to “time out” after trying to establish a threshold for a certain number (10,000) of iterations. In order to test whether this was the cause of the failure to find a threshold, we eliminated this limit (we also eliminated it in the time analysis above) and compared the results with those from our new algorithm. This empirical test of the robustness of the two methods showed that while the thresholding we applied using Otsu’s method was extremely robust (no failure detected on any sample independently of size or bit depth), the Irinato’s method had a variable but high rate of failure in establishing a threshold (Table 1). This result was also true with simulated artificial samples (not shown).

The methodology implemented by Irianto *et al*. to establish thresholds requires that two local maxima are determined in the histogram for the analyzed image and then a local minimum is established between them. While this method of establishing thresholds seems straight forward, when faced with noisy images of more heterogeneous cell populations *in situ*, rather than those obtained from cultured cells, the peaks and valleys in the histogram are not as easily detected and might not be a single pixel in width. This is likely to prevent local maxima from being determined. Irianto *et al*.’s method smoothed the histogram every time more than two peaks were detected. This further reduces the difference between peaks and valleys, and increases the width of the peaks beyond the single pixel expected by the algorithm, causing it to both not find peaks and fail to establish an appropriate threshold. By contrast, our algorithm utilizes MATLAB’s implementation of Otsu’s method, which utilizes the cumulative momentum of the histogram and discriminant analysis to obtain an automatic threshold in a non-recursive and robust fashion. Thus, our new method of quantifying chromatin condensation can more efficiently be used to generate data from images obtained from complex biological tissues, which can be compared between samples to more accurately reflect the biological processes occurring.

### Quantification of chromatin compaction agrees with standard staging during zebrafish spermatogenesis

Spermatogenesis is a well-defined biological process characterized by the formation of numerous haploid spermatozoa from diploid spermatogonial cells [18]. This systematic differentiation process involves discrete morphological changes including cytoplasmic, such as a reduction in cell size, and nuclear alterations, including extensive chromosome condensation; all of which have been qualitatively characterized using histological techniques in the zebrafish [19].

**Fig 2:**
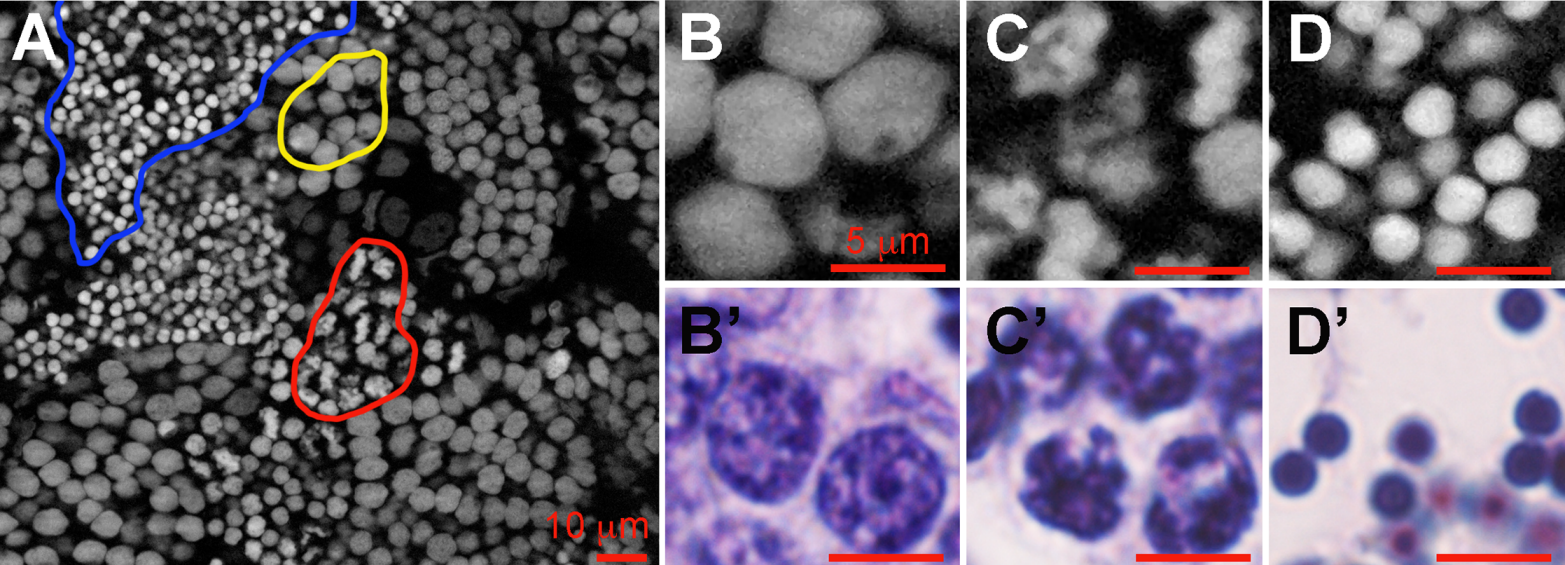
Representative images of chromatin compaction in developing spermatogonia. Images were obtained of histologically or DAPI stained tissue sections of adult zebrafish testes. (A-D) Representative fluorescent images of DAPI stained nuclei of B type spermatogonia (B), spermatocytes (C), and spermatids (D). Representative bright field images of B type spermatogonia (B’), spermatocytes (C’), and spermatids (D’) that have been stained with hematoxalin and eosin to characterize their basic cellular morphology. Scale bar in A is 10 μm, and in B-D’ is 5 μm.

In zebrafish spermatogenesis, diploid spermatogonia undergo several rounds of mitosis before entering meiosis and then spermiogenesis to differentiate into spermatozoa. Spermatogonia A include the presumed stem cells and early spermatogonia, and have a large round nucleus, two or three nucleoli, and very little heterochromatin [19]. This cell-type proceeds to differentiate into a spermatogonia B cell which, relative to its precursor, has an oval but shrunken nucleus that now has a slightly larger quantity of condensed chromatin and one or two nucleoli [19]. These features of spermatogonia B cells are presented in Fig 2. Spermatogonia progress to spermatocytes, which are meiotic cells and are associated with striking qualitative changes in the chromosome condensation. As depicted in Fig 2, the zygotene/pachytene/diplotene spermatocytes have rounder, denser nuclei compared with the type B spermatogonia and are enriched with condensing chromatin [19]. Completion of meiosis results in haploid spermatids characterized by an even more compact and condensed nuclear architecture [19]. Histologically, the spermatids continue to undergo spermiogenesis, which involves extreme compaction of the DNA so to fit within the tiny sperm head (Fig 2).

**Fig 3:**
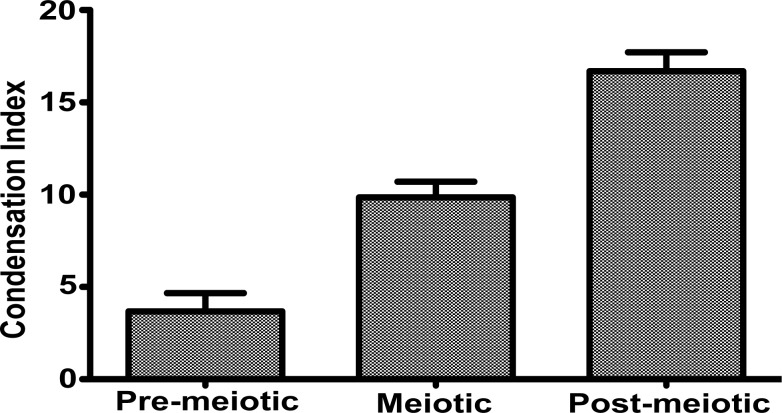
Quantification of the chromatin compaction index during spermatogenesis. The average compaction index of the DAPI stained nuclei of Spermatogonia B (n = 15 samples), spermatocytes (n = 20 samples), and spermatids (n = 15 samples) was quantified using the algorithm described in the materials and methods section. The histogram represents the average compaction index of the nuclei that were quantified in each cell type. Error bars are standard error. Analysis of variance followed by Bonfarroni’s multiple comparison test indicates that each bar is significantly different from all other samples (range of p = 0.01–0.001).

In order to quantify the microscopic image stacks, a simple ImageJ plugin was developed and utilized to segment, in 3-dimensions, the different testicular cell types according to morphology as described in Fig 2 (see materials and methods). These segmented images were subsequently analyzed using our improved algorithm in MATLAB (see materials and methods). Consistent with the qualitative histology data, we observed that each progressive stage of spermatogenesis is associated with a significant increase in the relative level of chromatin compaction (Fig 3). Thus, in addition to validating the improved algorithm as a tool to measure chromatin condensation in cells *in situ*, here we establish that this tool could be used to characterize the different stages of spermatogenesis.

### Quantification of chromatin de-condensation in regenerating amphibian cells

Urodele amphibians such as salamanders and newts have the amazing capacity to regenerate complete limbs. Although dramatic large-scale modifications have been qualitatively observed in the chromatin structure of regenerating cells [12], the morphology of the tissue has made it challenging to quantify these changes robustly and on a large-scale. In particular, regenerating cells have a low cytoplasmic to nucleus ratio; the nuclei are very close in proximity to each other, and are often irregular in shape. Thus, the available methodology to quantify chromatin compaction could not be effectively applied to the nuclei of these cells *in situ*.

**Fig 4:**
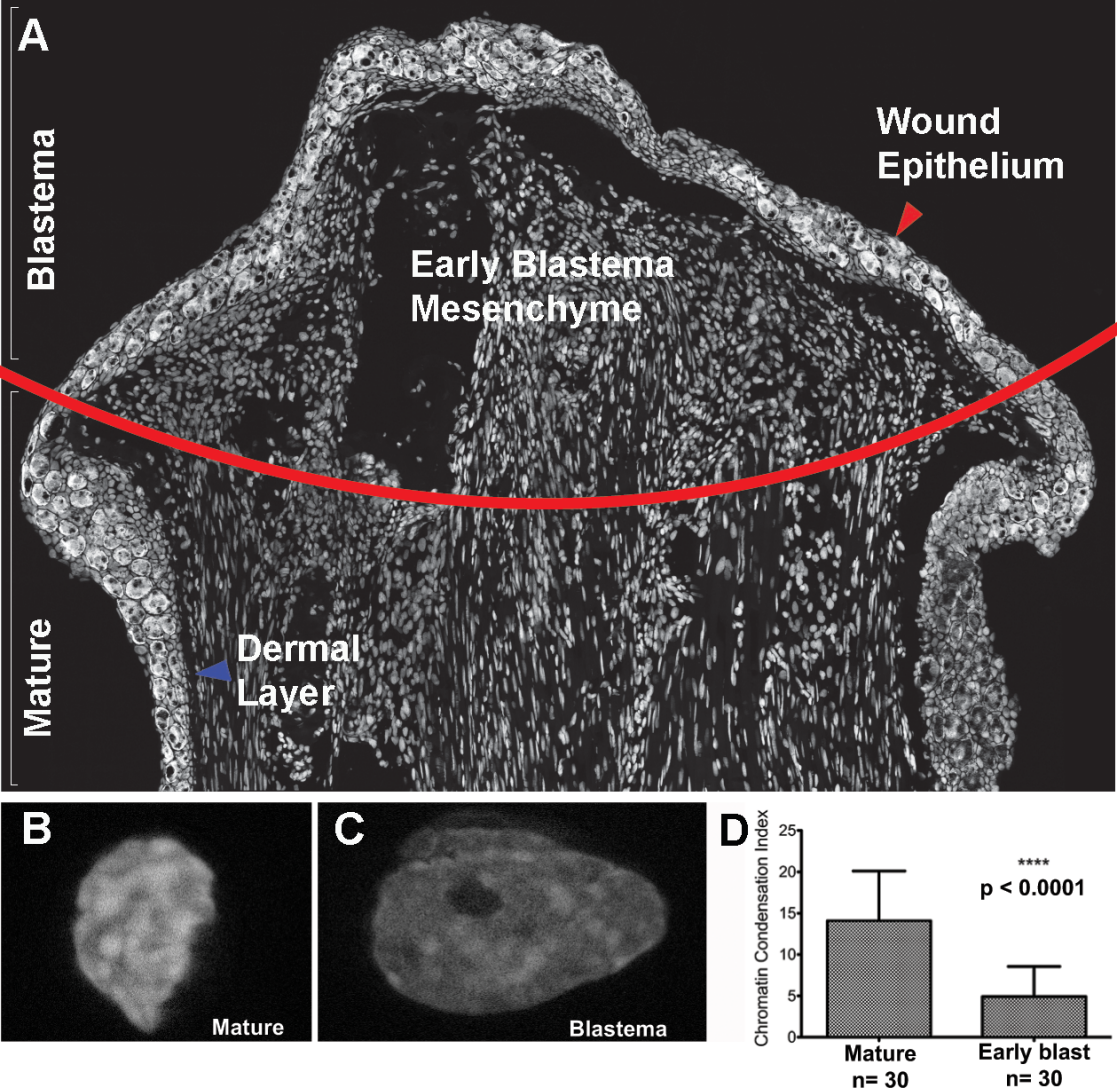
Use of the new quantification algorithm to characterize chromatin modifications in regenerating Axolotl limb cells. Fluorescent images were obtained of the DAPI stained nuclei in tissue sections of regenerating axolotl limbs at the early-blastema stage (A), and the chromatin compaction index was quantified. The red line indicates the amputation plane to indicate where the mature tissue ends and the blastema tissue begins, the red arrow points to the wound epithelium covering the blastema mesenchyme, and the blue arrow points to the dermal layer below the epidermis in the mature skin. Representative image of a DAPI stained nucleus of a mature (uninjured) cell in the dermis (B) and in the early regenerating blastema (C). (D) The chromatin compaction index was quantified as described in the materials and methods section and the average compaction index was plotted for mature (n = 30 samples) and regenerating (n = 30 samples) limb cells. Error bars are standard error. Analysis of variance was performed for statistical analysis (p<0.0001).

As represented in Fig 4, the sparsely positioned dermal cells of mature limb tissue can be visually described as exhibiting a highly compact chromatin structure constituted by numerous foci of intense DAPI staining (Fig 4B). This pattern of nuclear morphology is expected as it is characteristic of many differentiated cell types. In response to limb amputation, various biological processes are activated which culminate in the formation of a blastema, a population of regeneration-competent cells, at the site of injury (Fig 4A). This regeneration-specific structure is constituted by nearby mature somatic cells which migrate in, accumulate, and proliferate at the wounded site [20,21]; at early stages of regeneration this blastema is derived largely from cells of connective tissue origin [22,23]. The dense aggregate of early-stage blastema cells are visually distinct from somatic dermal cells in terms of their nuclear morphology–being larger and euchromatic in nature (compare Fig 4B and 4C). When analyzed with the Sosnik *et al*. algorithm, the chromatin of early bud blastema cells was found to be significantly less condensed relative to that of somatic dermal cells; verifying Hay’s (1959) observation that somatic cells undergo large-scale chromatin modifications when contributing to the blastema [12]. This opening of the chromatin is reminiscent of somatic reprogramming, whereby chromatin de-compaction accompanies the conversion of somatic cells back to a more pluripotent state (as reviewed in [8]). Connective tissue derived blastema cells exhibit plasticity in terms of their contribution to different cell-types in the regenerate [24,25]; therefore, this change in chromatin structure might play a role in the reprogramming of this mature cell type into a de-differentiated state.

Although it is beyond the scope of this study, this validated tool can be used to address various questions pertaining to regeneration. These include whether changes in chromatin architecture varies temporally and spatially in the blastema or even between the different cell-lineages which contribute to the blastema. Furthermore, this algorithm could be coupled with transcriptional analysis studies to provide insight into the functional consequences of chromatin de-compaction during regeneration.

## Conclusion

Here we describe an improved method for the quantification of chromatin compaction *in situ*. Unlike previously published methods, which either require the use of specialized visualization techniques such as fluorescence lifetime imaging [11], or are limited to quantifying images of homogeneous cellular samples [10], the Sosnik *et al*. algorithm presented here can be used with simple fluorescence microscopy data on an array of sample types, from different species, both *in vitro* and *in situ*. In addition, the presented algorithm allows rapid analysis of large datasets at high resolution (images as large as 2018 by 2048 pixels at 16-bits). Therefore, this method provides a standardized procedure with which to characterize chromatin condensation quantitatively for a plethora of different cell types and, in so doing, improve our general understanding of chromatin-related cellular state and functionality. Furthermore, in terms of regeneration, we have shown that the nuclei of blastema cells exhibit a significant, large-scale opening of their chromatin, relative to mature dermal cells, in the axolotl. These changes are reminiscent of somatic reprogramming and may facilitate the plasticity of blastema cells as they contribute to the regenerate.

## Supporting information

S1 Fig Graphical representation of our new algorithm for chromatin condensation quantitative analysis. (PDF)

S2 Fig Graphical representation of the folder and file architecture utilized as input by the MATLAB routine. (PDF)

S1 Text Text file of.m file. (DOCX)

S2 Text Text file of.ijm file. (DOCX)
