# Supplementary figures and images for: A new and improved algorithm for the quantification of chromatin condensation from microscopic data shows decreased chromatin condensation in regenerating axolotl limb cells
Source: PLoS One. 2017 Oct 12;12(10):e0185292. doi: 10.1371/journal.pone.0185292 (PMC5638231; doi:10.1371/journal.pone.0185292)

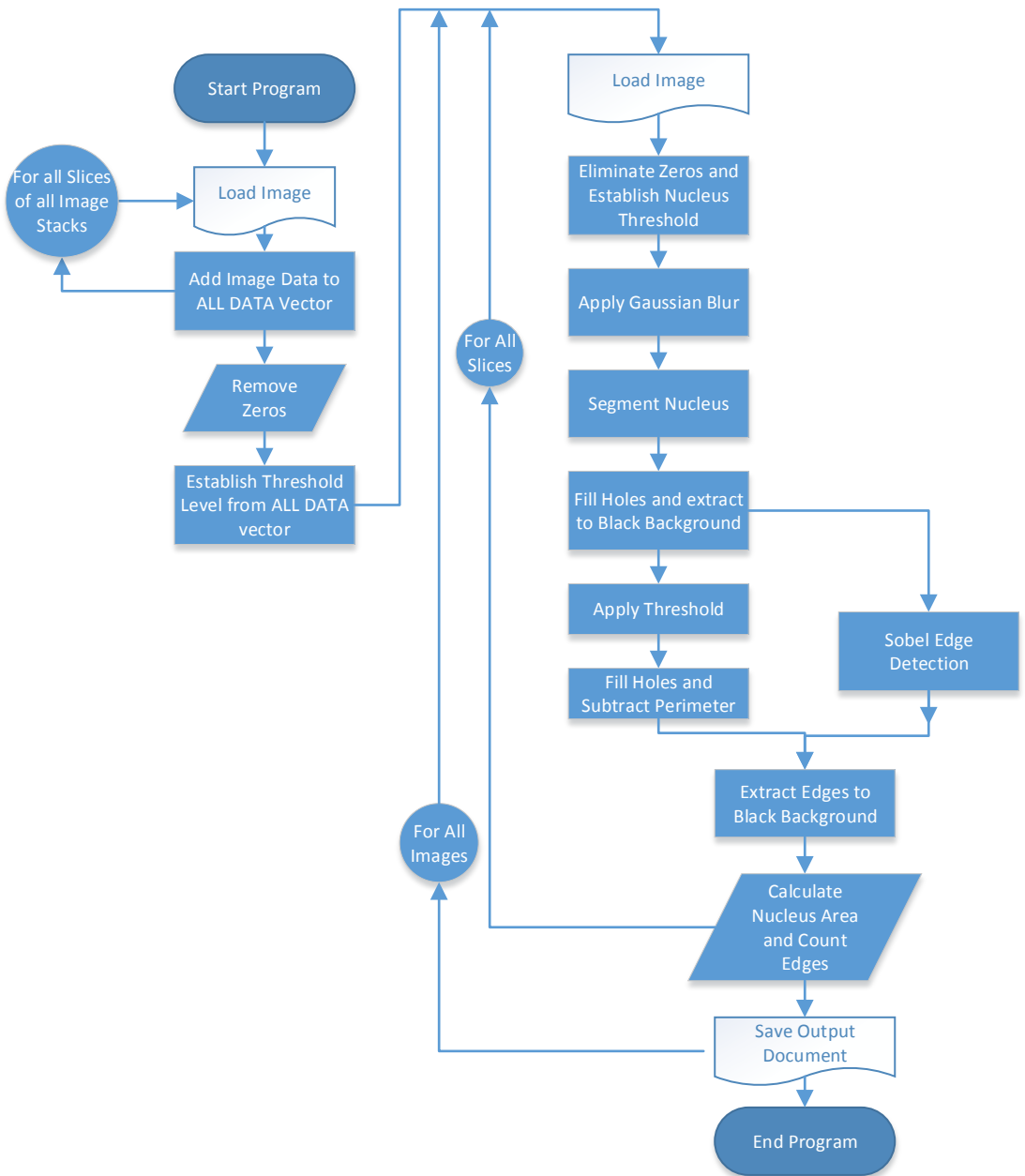

Supplement: S1 Fig — (PDF) [file pone.0185292.s001.pdf]

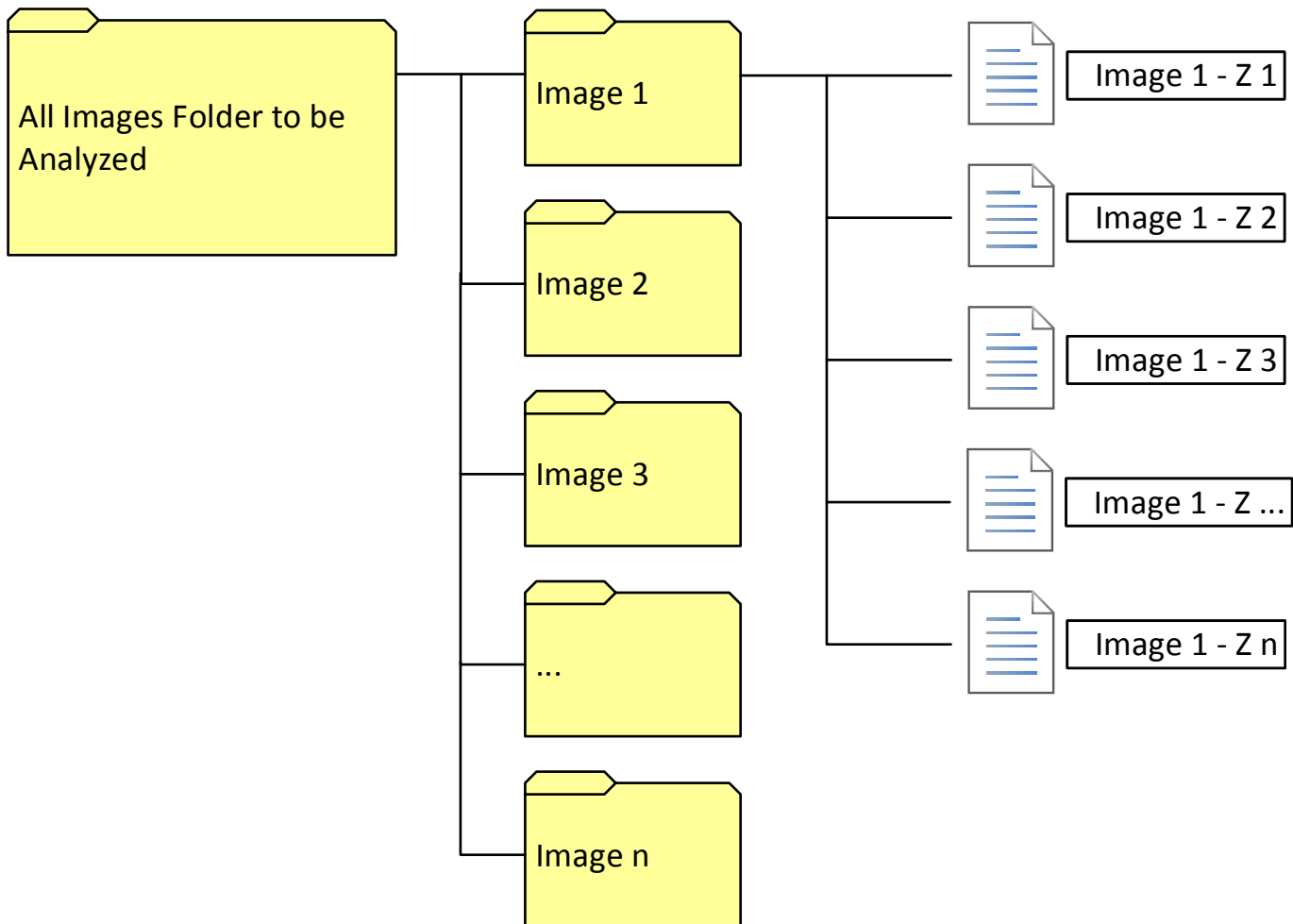

Supplement: S2 Fig — (PDF) [file pone.0185292.s002.pdf]
