# Supplementary material for: A new and improved algorithm for the quantification of chromatin condensation from microscopic data shows decreased chromatin condensation in regenerating axolotl limb cells
Source: PLoS One. 2017 Oct 12;12(10):e0185292. doi: 10.1371/journal.pone.0185292 (PMC5638231; doi:10.1371/journal.pone.0185292)
Supplement: S1 Text — (DOCX) [file pone.0185292.s003.docx]

%% ChromCond.m

% created by Prof. Julian Sosnik for Prof. Catherine McCusker

% Copyright (C) 2017 Julian Sosnik

%

%This work is distributed under the Modified BSD license. For full license

%text see lines 244 to 268 or go to https://opensource.org/licenses/BSD-3-Clause

%

%This program performs chromatin condensation quantification based on the

%algorithm published by Irianto, Lee and Knight (2014)

%doi:10.1016/j.medengphy.2013.09.006

%

% It performs chromatin condensation analysis of

% consecutive Z slices from a stack of images.

% It can also handles multiple image folders. Place all the folders containing

% the individual z-stacks of images to be analyzed in a single master folder.

% Keep the subfolders containing the individual Z slices (as tiff files) inside the master

% folder.

%

% Many parameters within the analysis can be modified if desired. To do so

% simply type edit ChomCond.m in the Command Window and read the comments

% starting on line 26.

clear;

clc

%% Analysis options

% to change the sigma of the Gaussian filter make sigma ~= 1

% to skip the Gaussian filter completely make sigma = 0 NOT RECOMENDED

% SobelThresh is the threshold for the Sobel edge algorithm (for auto = 0)

% n is the level of erosion to apply. For none, set to 0.

% thresh is the number of threshold bins to apply to the segmented nuclei

% set to 0 for automatic thresholding

% threshLimit selects the smallest bin from the thresholded image to keep

sigma = 1;

if sigma

h = fspecial('gaussian', 3, sigma);

end

SobelThresh = 0.00;

n = 2;

thresh = 0;

threshLimit = 2;

goback = pwd;

count = 0;

%% Main routine starts here

directory = uigetdir;

cd (directory);

dirnames = dir(directory);

dirname = dirnames([dirnames.isdir]); % Generates list of directories

numdirs = numel(dirname);

for i = numdirs:-1:1

if dirname(i).name(1) == '.' % Eliminate system directories

dirname(i) = [];

end

end

%% Count the number of files to be analyzed

for i = 1:numel(dirname)

cd(dirname(i).name);

filenamess = dir('*.tif'); % Make list of tif files in directory

filenamess = filenamess(logical(abs([filenamess.isdir]-1))); % make sure is a file

ff = numel(filenamess);

for ii = ff:-1:1

if filenamess(ii).name(1) == '.'

filenamess(ii) = []; % Eliminate non-image files

end

end

count = count + numel(filenamess); % Count total number of files

cd ..

end

stru{count} = []; % Generate a structure to hold the images

%% Generate a cell array containing all the images to calculate threshold

k = 0;

for i = 1:numel(dirname) % Cycle all the directories

cd(dirname(i).name);

filenamess = dir('*.tif'); % list all tiff files

ff = numel(filenamess);

for ii = ff:-1:1

if filenamess(ii).name(1) == '.'

filenamess(ii) = []; % eliminate non-image files

end

end

for j = 1:numel(filenamess) % cycle through all the images

k = k+1;

T0 = imread(filenamess(j).name); % read image

T0 = T0(logical(T0)); % eliminate zeros (make dense)

stru{k} = T0; %store dense image in structure

end

cd ..

end

T1 = cat(1,stru{:}); % concatenate all images into single vector

clear stru

%% calculate main threshold

T = multithresh(T1);

if thresh == 0

T2 = multithresh(T1(T1>T));

else

T2 = multithresh(T1(T1>T),thresh);

end

clear T1 T0

%% Initiates main loop directory by directory

for i = 1:numel(dirname)

cd (dirname(i).name);

filenames = dir('*.tif'); %generates list of files to process

ff = numel(filenames);

for ii = ff:-1:1

if filenames(ii).name(1) == '.'

filenames(ii) = []; % eliminate non-image files

end

end

output = strcat(dirname(i).name, '.csv');

out1 = output(1:end-4);

s = numel(filenames); %generate empty results matrices

AreaList = zeros(s,1);

EdgeCountList = zeros(s,1);

EdgeRatioList = zeros(s,1);

%% Start of the file analysis loop (file by file in directory)

for j = 1:numel(filenames)

try

%% Analysis of the image begins here

I0 = imread(filenames(j).name);

% generate dense matrix from I0

[r, c, dense1] = find(I0);

% thresholding value for I0

T = multithresh(dense1);

% Gaussian filter (eliminates high frequency noise)

I1 = I0;

if sigma > 0

I1 = imfilter(I1,h);

end

%Threshold application to I1

I2 = imquantize(I1,T);

I2(I2==1)=0;

I2 = logical(I2);

%Hole-filling algorithm

I3 = imfill(I2,'holes');

%Extract the nucleus from the original image to a black background

[row,column] = find(I3);

[sizerow,sizecolumn] = size(I3);

I4 = zeros(sizerow,sizecolumn);

for k = 1:length(row)

R = row(k);

C = column(k);

I4(R,C) = I0(R,C);

end

%SOBEL edge detection application

if SobelThresh == 0

I5 = edge(I4,'sobel');

else

I5 = edge(I4, 'sobel',SobelThresh);

end

%Threshold application to I4

I6 = imquantize(I4,T2);

I6(I6<threshLimit)=0;

I6 = logical(I6);

%Hole-filling algorithm

I7 = imfill(I6,'holes');

%Perimeter subtraction (n times)

I8 = I7;

if n ~= 0

for k = 1:n

I9 = bwperim(I8);

I8 = I8-I9;

I8 = logical(I8);

end

end

%Extract the SOBEL edge inside the nucleus into a black background

[row,column] = find(I8);

[sizerow,sizecolumn] = size(I8);

I10 = zeros(sizerow,sizecolumn);

for k = 1:length(row)

R = row(k);

C = column(k);

I10(R,C) = I5(R,C);

end

I10 = logical(I10);

%Nucleus area

[row,column] = find(I3);

Area = length(row);

AreaList(j,1) = Area;

%Edge count

edgeCount = sum(sum(I10));

EdgeCountList(j,1) = edgeCount;

%Edge density (i.e. chromatin condensation parameter)

edgeRatio = (edgeCount/Area)*100;

EdgeRatioList(j,1) = edgeRatio;

%%Error resolution

catch

AreaList(j,1) = 0;

EdgeCountList(j,1) = 0;

EdgeRatioList(j,1) = 0;

end

end

%% results are compiled and saved

len = length(AreaList);

results = zeros(len,4);

for k= 1:len

results(k,1) = k;

results(k,2) = AreaList(k);

results(k,3) = EdgeCountList(k);

results(k,4) = EdgeRatioList(k);

end

resu = results.';

cd ..

%saving results

fileID = fopen(output, 'w');

fprintf(fileID, 'file, Area, Edge, Ratio\n');

fprintf(fileID, '%3d, %8d, %6d, %3.8f\n', resu);

fclose all;

end

cd(goback)

%% Full License Text:

% Redistribution and use in source and binary forms, with or without modification,

% are permitted provided that the following conditions are met:

%

% 1. Redistributions of source code must retain the above copyright notice,

% this list of conditions and the following disclaimer.

%

% 2. Redistributions in binary form must reproduce the above copyright notice,

%this list of conditions and the following disclaimer in the documentation

%and/or other materials provided with the distribution.

%

% 3. Neither the name of the copyright holder nor the names of its contributors

%may be used to endorse or promote products derived from this software

%without specific prior written permission.

%

% THIS SOFTWARE IS PROVIDED BY THE COPYRIGHT HOLDERS AND CONTRIBUTORS "AS IS"

%AND ANY EXPRESS OR IMPLIED WARRANTIES, INCLUDING, BUT NOT LIMITED TO, THE IMPLIED

% WARRANTIES OF MERCHANTABILITY AND FITNESS FOR A PARTICULAR PURPOSE ARE DISCLAIMED.

%IN NO EVENT SHALL THE COPYRIGHT HOLDER OR CONTRIBUTORS BE LIABLE FOR ANY

%DIRECT, INDIRECT, INCIDENTAL, SPECIAL, EXEMPLARY, OR CONSEQUENTIAL DAMAGES

%(INCLUDING, BUT NOT LIMITED TO, PROCUREMENT OF SUBSTITUTE GOODS OR SERVICES;

%LOSS OF USE, DATA, OR PROFITS; OR BUSINESS INTERRUPTION) HOWEVER CAUSED

%AND ON ANY THEORY OF LIABILITY, WHETHER IN CONTRACT, STRICT LIABILITY, OR

%TORT (INCLUDING NEGLIGENCE OR OTHERWISE) ARISING IN ANY WAY OUT OF THE USE

%OF THIS SOFTWARE, EVEN IF ADVISED OF THE POSSIBILITY OF SUCH DAMAGE.
