# Supplementary material for: A new and improved algorithm for the quantification of chromatin condensation from microscopic data shows decreased chromatin condensation in regenerating axolotl limb cells
Source: PLoS One. 2017 Oct 12;12(10):e0185292. doi: 10.1371/journal.pone.0185292 (PMC5638231; doi:10.1371/journal.pone.0185292)
Supplement: S2 Text — (DOCX) [file pone.0185292.s004.docx]

// Created by Prof. Julian Sosnik for Prof. Catherine McCusker's Lab

// evolved from a macro created by Dr. Matheus Viana / Dr. Susanne Rafelski

//

//This work is distributed under the Modified BSD license (https://opensource.org/licenses/BSD-3-Clause)

//

///////////////////////////////////////////////////////////////////////////

//This macro opens a file, let's you select an area and then creates a cropped version

// of your image (or stack, or Hyperstack) that contains only the portion

// of the original image that you selected...

// In the case of multiple channels, it generates sub-folders

// for each of the channels and stores the images there

//////////////////////////////////////////////////////////////////////////

run("Open...");

// gather file name and path for the original image

path = getInfo("image.directory");

name = getTitle();

l = lengthOf(name);

newName = substring(name,0,l-4);

getDimensions(width, height, channels, slices, frames);

File.makeDirectory(path+newName); //creates folder to save the output files

//select area to crop into new image

waitForUser("Select area to crop and hit ok");

/// Add selection to ROI manager to use later

roiManager("reset");

setOption("Show All", false)

if (selectionType() == -1){ // Checks to see if there is a selection made to make an ROI

makeRectangle(0, 0, width, height); // If there is not one, it makes one with the dimensions of the whole image

}

roiManager("add");

roiManager("remove slice info");

Roi.getBounds(roix, roiy, roiWidth, roiWeight);

setBatchMode(true); // runs in background to accelerate process

setBatchMode("hide");

chan = channels;

if (chan>1){

run("Split Channels");

}

for (ii=1; ii<=chan; ii++){

File.makeDirectory(path+newName+"/C"+ii); //creates sub-folder to save the output files for each channel

if (chan>1){

orig = "C"+ii+"-"+name; // now can refer to that image as "orig"

} else {

orig = name;

}

selectImage(orig); //select the image from the channel ii

// gather information about the original image

info = getMetadata("Info");

getDimensions(width, height, channels, slices, frames);

getVoxelSize(Vwidth, Vheight, depth, unit);

fps = Stack.getFrameInterval();

//make a new image z-stack

newImage("C"+ii+"-"+newName+"_cropped", "16-bit Black", roiWidth, roiWeight, slices);

final = getImageID; //now can refer to this new z-stack as "final" and go back and forth between orig and final

setMetadata("Info", info);

run("Properties...", "channels="+channels+" slices="+slices+" frames="+frames+" unit="+unit+" pixel_width="+Vwidth+" pixel_height="+Vheight+" voxel_depth="+depth+" frame=["+fps+" sec]");

Stack.setFrameRate(fps);

//paste selection into the new stack frame by frame

for (n=1; n<=slices; n++) {

selectImage(orig);

setSlice(n);

roiManager("select", 0); // Apply the ROI from the original image

run("Copy");

selectImage(final);

setSlice(n);

run("Paste");

}

selectImage(orig);

close();

selectImage(final);

namefinal = "C"+ii+"-"+newName+"_cropped";

save(path+newName+"/"+namefinal+".tif"); //saves cropped image

//separates the slices into individual images

run("Stack to Images");

for (i=1; i<=slices; i++){

j = IJ.pad(i, 4);

imag = namefinal+"-"+j;

selectImage(imag);

na = getTitle();

save(path+newName+"/C"+ii+"/"+na+".tif");

close();

}

}

//// Cleanup

roiManager("reset");

selectWindow("ROI Manager");

run("Close");

////// THE CODE ENDS HERE, NEXT LINE IS SO YOU KNOW WHEN THE PROGRAM IS DONE RUNNING

waitForUser("Done"); // Exit message
